# The methylome of the marbled crayfish links gene body methylation to stable expression of poorly accessible genes

**DOI:** 10.1186/s13072-018-0229-6

**Published:** 2018-10-04

**Authors:** Fanny Gatzmann, Cassandra Falckenhayn, Julian Gutekunst, Katharina Hanna, Günter Raddatz, Vitor Coutinho Carneiro, Frank Lyko

**Affiliations:** 1 Division of Epigenetics, DKFZ-ZMBH Alliance, German Cancer Research Center, Im Neuenheimer Feld 580, 69120 Heidelberg, Germany; 2 Faculty of Biosciences, University of Heidelberg, Im Neuenheimer Feld 234, 69120 Heidelberg, Germany

## Abstract

**Background:**

The parthenogenetic marbled crayfish (*Procambarus virginalis*) is a novel species that has rapidly invaded and colonized various different habitats. Adaptation to different environments appears to be independent of the selection of genetic variants, but epigenetic programming of the marbled crayfish genome remains to be understood.

**Results:**

Here, we provide a comprehensive analysis of DNA methylation in marbled crayfish. Whole-genome bisulfite sequencing of multiple replicates and different tissues revealed a methylation pattern that is characterized by gene body methylation of housekeeping genes. Interestingly, this pattern was largely tissue invariant, suggesting a function that is unrelated to cell fate specification. Indeed, integrative analysis of DNA methylation, chromatin accessibility and mRNA expression patterns revealed that gene body methylation correlated with limited chromatin accessibility and stable gene expression, while low-methylated genes often resided in chromatin with higher accessibility and showed increased expression variation. Interestingly, marbled crayfish also showed reduced gene body methylation and higher gene expression variability when compared with their noninvasive mother species, *Procambarus fallax.*

**Conclusions:**

Our results provide novel insights into invertebrate gene body methylation and its potential role in adaptive gene regulation.

**Electronic supplementary material:**

The online version of this article (10.1186/s13072-018-0229-6) contains supplementary material, which is available to authorized users.

## Background

The marbled crayfish (*Procambarus virginalis*) represents a novel freshwater crayfish species that emerged in the German aquarium trade in 1995 [1]. Marbled crayfish reproduce by apomictic parthenogenesis, thus producing large numbers of genetically identical offspring [2–4]. It is assumed that *P. virginalis* originated from a very recent evolutionary macromutation in the Florida slough crayfish *Procambarus fallax* [5, 6]. Comparative whole-genome sequencing of a diverse set of animals from various sources has shown that the global population can be considered a single genetic clone with negligible genetic variation [7].

Despite their genetic homogeneity, marbled crayfish have successfully invaded and colonized a variety of habitats in subtropical and temperate regions [8, 9]. This is exemplified by the rapid propagation of marbled crayfish on Madagascar, where the animals have increased their distribution area 100-fold over the past 10 years [7]. Of note, the genetic homogeneity of the population precludes the selection of genetic variants as an explanation for rapid adaptation. As such, it is important to understand epigenetic regulation in marbled crayfish.

DNA methylation represents a conserved and well-established epigenetic modification [10–12]. It is mediated by the family of DNA methyltransferases (DNMTs) which catalyze the methylation of genomic cytosine residues in a wide range of organisms and provide an important toolkit for epigenetic regulation [13]. Two previous studies have used capillary electrophoresis and mass spectrometry to demonstrate the presence of DNA methylation in marbled crayfish [4, 6]. In addition, changes in global DNA methylation levels have been linked to phenotypic variants [4]. However, information about the DNA methylation toolkit, the patterning of DNA methylation and its potential function has been lacking.

Single-base resolution methylation maps have been generated for a large variety of species and have shown a surprising diversity of DNA methylation patterns in the animal kingdom [14–16], ranging from almost ubiquitous methylation to low levels or no methylation. Also, different species can show methylation in distinct subgenomic compartments, including promoters, repetitive elements and gene bodies [14, 15]. Interestingly, gene body methylation is often associated with housekeeping genes [17, 18].

The function of gene body methylation remains to be fully understood [19, 20]. The modification has been linked to various aspects of gene regulation, including transcriptional elongation, mRNA splicing, chromatin structure and the suppression of cryptic intragenic promoters in transcribed chromatin [21–24]. In invertebrates, the preferential methylation of highly conserved genes with housekeeping functions and their moderate but stable expression similarly suggests a regulating function for gene expression, possibly through the suppression of transcriptional noise or expression variation [17, 18, 25, 26]. It has also been shown that conflicting chromatin states, determined by the absence of active histone marks and presence of repressive histone marks, are associated with high levels of transcription noise in actively transcribed genes [27]. However, a clear mechanistic understanding of the role of gene body methylation in gene expression variability remains lacking.

We have now used whole-genome bisulfite sequencing to establish high-resolution methylation maps of *P. virginalis* from several independent animals and tissues. We also performed RNA-seq and chromatin accessibility assay sequencing (ATAC-seq) to integratively analyze the effect of gene body methylation on chromatin accessibility states and gene expression. The results reveal a DNA methylation pattern that is characterized by tissue-invariant gene body methylation of housekeeping genes. While gene body methylation was negatively associated with chromatin accessibility, we also found that active genes with variable expression were less accessible than stably expressed genes. Comparing gene body methylation levels and gene expression variation levels in the marbled crayfish and its parent species *P. fallax*, we observe overall lower gene body methylation levels and higher gene expression variation levels in the marbled crayfish. Together, these findings establish the methylome of an emerging invasive animal and provide novel insight into invertebrate gene body methylation.

## Results

### Identification of a conserved DNA methylation system in marbled crayfish

**Fig. 1:**
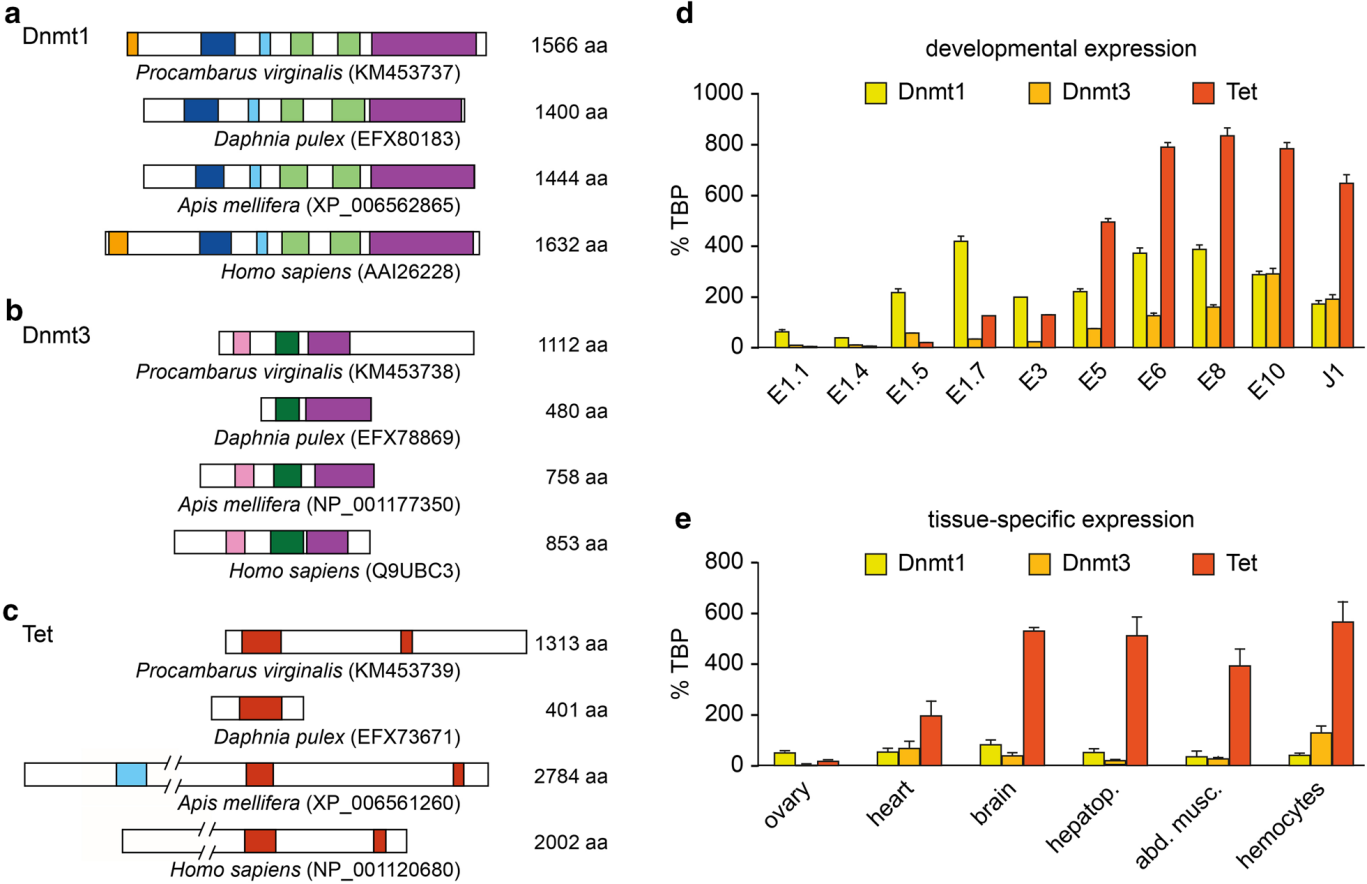
Conservation of Dnmt1, Dnmt3 and Tet in marbled crayfish. Genome annotation revealed the presence of a DNA methylation system consisting of single homologs for Dnmt1 (**a**), Dnmt3 (**b**) and Tet (**c**), respectively. Shown are comparisons of virtually translated protein sequences with three reference organisms: *Daphnia pulex*, *Apis mellifera* and *Homo sapiens*. Numbers in brackets represent accession numbers. Conserved domains are shown as colored boxes. Dnmt1: orange—DMAP1-binding domain, dark blue—replication foci domain, light blue—CXXC zinc finger domain, light green—bromo adjacent homology domain, purple—catalytic domain. Dnmt3: pink—PWWP domain, dark green—zinc finger domain, purple—catalytic domain. Tet: red—catalytic domain, light blue—CXXC zinc finger domain. **d** Expression of Dnmt1, Dnmt3 and Tet during marbled crayfish development. mRNA expression levels are indicated relative to the *TBP* (TATA-box-binding protein) housekeeping gene. Bars indicate standard deviations from at least three independent measurements. E: embryonic stages; J: juvenile stages. **e** mRNA levels of Dnmt1, Dnmt3 and Tet in various adult marbled crayfish tissues (*hepatop.* hepatopancreas, *abd. musc.* abdominal musculature)

We have recently assembled the transcriptome of the marbled crayfish and also established a draft assembly of the complete genome sequence [7]. Genome annotation identified single crayfish homologs for Dnmt1, Dnmt3 and a Tet hydroxymethylase [13]. Virtual translation of the corresponding transcripts produced protein sequences with robust sequence conservation to functionally characterized honeybee and human homologs. A more detailed analysis of the predicted crayfish Dnmt1 homolog revealed a protein with a length of 1566 amino acids, which contained all the known Dnmt1 protein domains in the correct order (Fig. 1a). Furthermore, our analysis of the predicted marbled crayfish Dnmt3 protein identified a protein with 1112 amino acids with the known Dnmt3 protein domains (Fig. 1b). We also investigated the predicted crayfish Tet enzyme. This revealed a protein of 1313 amino acids with substantial sequence homology to honeybee and human Tet enzymes, including two conserved oxygenase domains (Fig. 1c). Together, these findings suggest the presence of a conserved DNA methylation and demethylation system in marbled crayfish.

To confirm the expression of the marbled crayfish DNA methylation system, we used qRT-PCR analysis of various developmental stages and dissected tissues from adult animals. Based on published data from *Daphnia pulex* [28] and an evaluation of five different housekeeping genes (Additional file 1), TATA-box-binding protein (TBP) mRNA was used as an internal reference. The results showed low mRNA levels for all three genes in early embryonic stages. Dnmt1 became strongly upregulated in embryonic stage 1.5, while Dnmt3 expression continuously increased during embryogenesis (Fig. 1d). Tet mRNA levels became strongly increased during mid-embryogenesis and remained high in juveniles (Fig. 1d). In adult tissues, Dnmt1 was stably expressed at moderate levels (Fig. 1e), which is consistent with a general maintenance methyltransferase function of the enzyme. The expression pattern of Dnmt3 appeared to be more tissue specific, with the highest level in hemocytes and lowest level in the ovary (Fig. 1e). Tet expression was high in most tissues, but low in the ovary (Fig. 1e). Together, these data show that the components of the DNA methylation system are dynamically expressed during marbled crayfish development and in adult tissues.

### Characterization of the marbled crayfish methylome

**Fig. 2:**
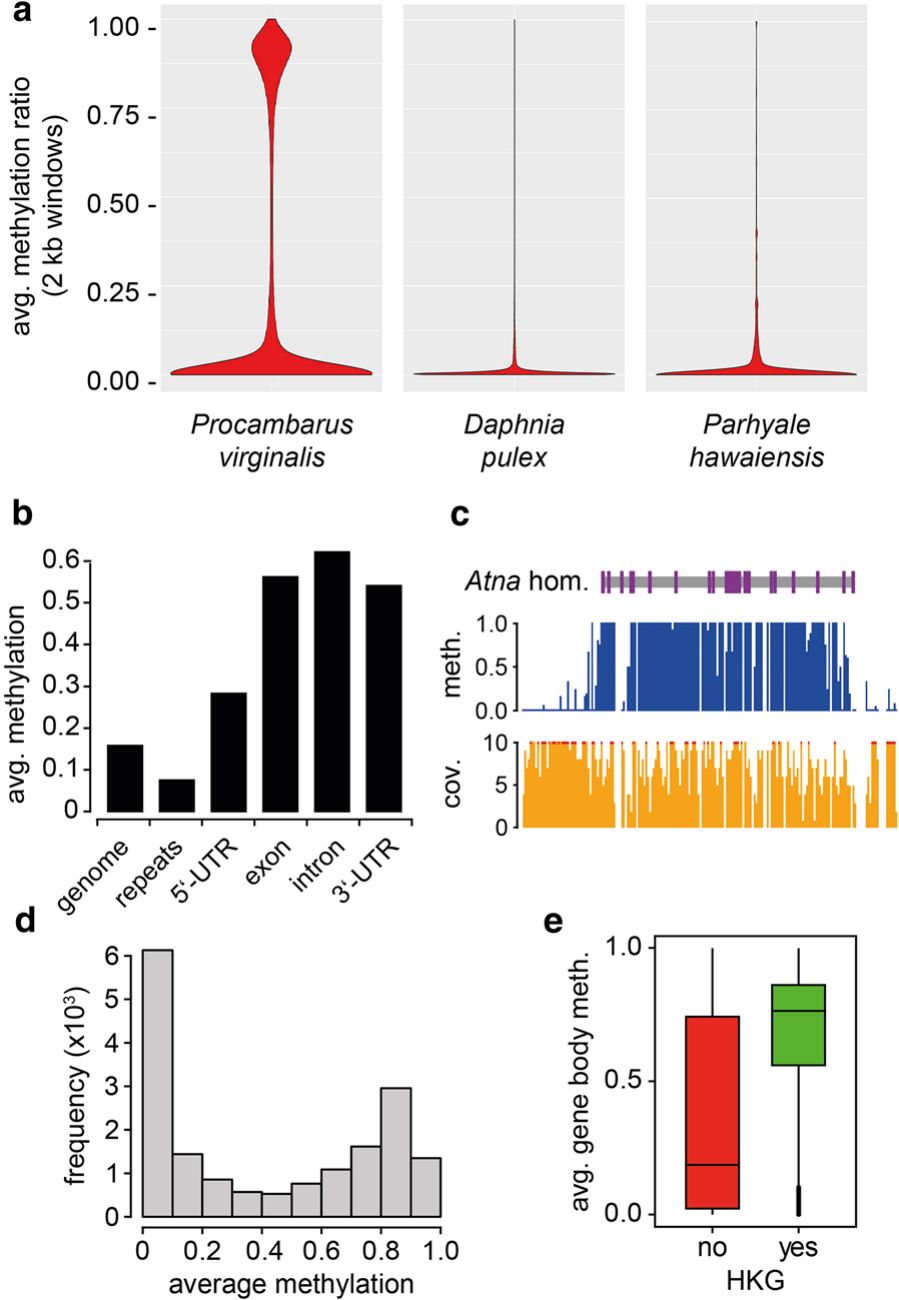
Characterization of the marbled crayfish methylome. **a** Comparative analysis of known crustacean methylomes. Violin plots show average CpG methylation levels of 2-kb sliding windows. **b** Methylation levels of the genome and of predicted gene features. **c** Representative Genome Browser track for a methylated gene, showing methylation ratio (blue) and coverage (orange). Red dots denote coverages > 10 ×. **d** Histogram showing the frequencies of average gene body methylation levels in bins of 0.1. **e** Boxplots showing the distribution of methylation ratios for non-housekeeping genes (red) compared with housekeeping genes (green)

We used whole-genome bisulfite sequencing to characterize methylation patterns at single-base resolution. Sequencing of hepatopancreas DNA at 9.8 × genome coverage (Additional file 2) uncovered a methylation pattern that was CpG specific, bimodal and symmetric (Additional file 3) and thus recapitulates key hallmarks of other known animal methylomes [14, 15]. A comparative analysis in 2-kb sliding windows (Fig. 2a) revealed that the marbled crayfish methylome showed substantially more highly methylated windows than other known crustacean methylomes [29, 30]. This suggests that the marbled crayfish genome is relatively highly methylated.

We also quantified CpG methylation levels for various subgenomic features, such as repeats, exons and introns. This revealed that repeats were relatively hypomethylated (Fig. 2b), while methylation was strongly enriched at gene bodies (Fig. 2b, c). More specifically, average methylation levels were found slightly increased over the genome average in 5’-UTRs and more strongly increased in exons, introns and 3’-UTRs (Fig. 2b). Interestingly, gene body methylation showed a bimodal distribution, with distinct populations of low-methylated and high-methylated genes (Fig. 2d). Further analysis revealed that methylation preferentially targets long, CpG-poor and evolutionarily conserved genes (Additional file 4). These characteristics represent defining features of housekeeping genes, and indeed, methylation levels of housekeeping genes were strongly elevated when compared with other genes (Fig. 2e). Together, our findings thus suggest that DNA methylation in marbled crayfish is enriched at gene bodies and identify housekeeping genes as an important methylation target.

### The marbled crayfish methylome is largely tissue invariant

**Fig. 3:**
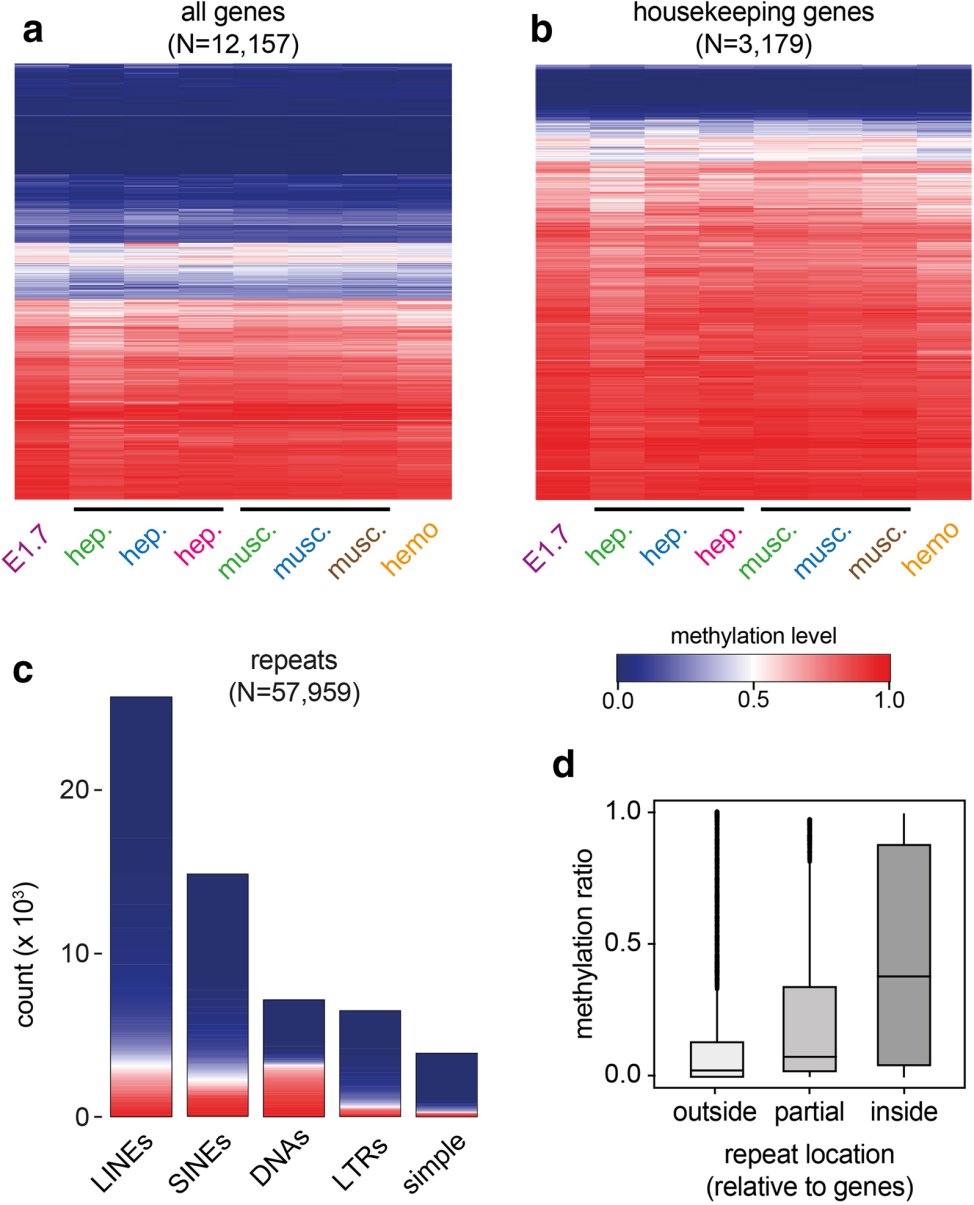
Comparative analysis of gene body and repeat methylation patterns from different developmental stages, tissues and animals. **a** Comparative analysis of gene body methylation patterns. The heatmap shows average gene body methylation levels in eight independent samples (columns). Methylation levels are indicated on a scale from 0 (blue) to 1 (red). Only genes with sufficient coverage in all eight samples are shown. E1.7: stage 1.7 embryos, hep.: hepatopancreas, musc.: abdominal musculature, hemo: hemocytes. Colors denote individual animals. **b** Parallel analysis of housekeeping genes. **c** Methylation of the most frequent repeat classes. LINEs (long interspersed nuclear elements): *N* = 25,622, SINEs (short interspersed nuclear elements): *N* = 14,821, DNAs (DNA transposons): *N* = 7144, LTRs (long terminal repeats): *N* = 6483, simple (simple repeats): *N* = 3889. **d** Location-dependent methylation of repeats

To further characterize DNA methylation in marbled crayfish, we performed whole-genome bisulfite sequencing on DNA samples from seven additional animals and tissues (Additional file 2). Our analysis included two distinct adult tissues (hepatopancreas and abdominal musculature), each from three independent animals and from various sources (laboratory stocks and wild catches). In addition, we also included single replicates from early embryos (stage 1.7) and a third adult tissue (hemocytes). Genome coverage ranged from 9 × to 23 × (Additional file 2), thus ensuring sufficient analytical power. Strikingly, a comparative analysis of all eight methylomes failed to reveal tissue-specific or developmental stage-specific changes (Fig. 3a). This was also confirmed by a Wilcoxon rank-sum test, which did not identify any significantly differentially methylated genes between abdominal musculature and hepatopancreas. A specific analysis of housekeeping genes confirmed their pronounced methylation and again suggested that the marbled crayfish methylome is largely tissue invariant (Fig. 3b). It remains possible that a subset of variably methylated genes show moderate context-dependent methylation changes, but a substantially greater number of samples will be required for their identification [31].

Further analysis also showed that the majority of repetitive elements was unmethylated, while some minor repeat classes, such as DNA transposons and TcMar-Tigger elements, showed higher methylation levels (Fig. 3c, Additional file 5). On the genome level, repeat methylation appeared strongly associated with gene body methylation, as repeats outside of genes had lower methylation levels compared to repeats that were located within genes (Fig. 3d). Of note, repeat methylation again appeared largely invariant between different tissues (Additional file 5), which is consistent with the methylation patterns observed for genes.

### Gene body methylation, chromatin accessibility and gene expression variability

To investigate potential gene regulatory functions of DNA methylation in marbled crayfish, we integrated our methylation datasets with RNA-seq datasets that were obtained from three independent hepatopancreas and abdominal musculature samples, respectively (Additional file 6). The results showed a parabolic correlation between gene body methylation and gene expression levels, with the highest and lowest expression ranks being relatively undermethylated (Additional file 7). These results are similar to findings originally made in plants [22, 32]. For further insight, we also addressed the relationship between DNA methylation and chromatin accessibility. For this purpose, we used ATAC-seq [33] to generate high-resolution chromatin accessibility maps that could be analyzed together with DNA methylation maps. ATAC-seq was successfully established for marbled crayfish hemocytes (Additional file 8), which are isolated cells that are suitable for ATAC-seq analysis (Additional file 9). We also generated RNA-seq data from hemocytes (Additional file 10) and integrated the WGBS data from hemocytes into our analysis.

**Fig. 4:**
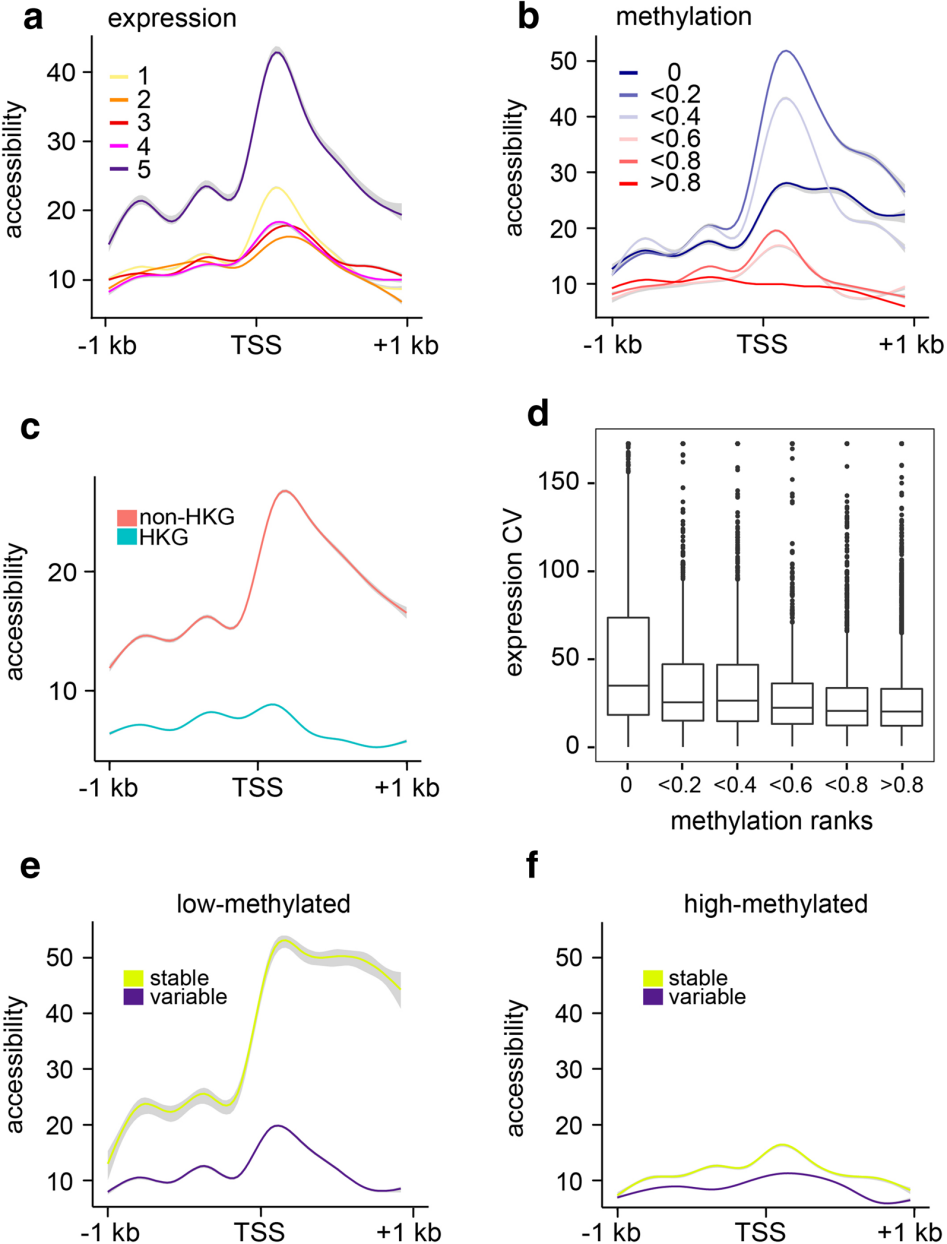
DNA methylation, chromatin accessibility and gene expression variability. **a** ATAC signals around transcription start sites are shown for gene expression quintiles. **b** ATAC signals around transcription start sites are shown for unmethylated genes and for gene body methylation quintiles. **c** Metagene plot for chromatin accessibility around the transcription start site for housekeeping genes and non-housekeeping genes. **d** Correlation between DNA methylation and gene expression variation in hemocytes. Methylation rank 0 represents completely unmethylated genes. **e** ATAC signals for low-methylated (methylation level < 0.4) and **f** high-methylated (methylation level > 0.4) genes, and gene sets with different expression variability (stable: variability quintile 1 with low expression variability; variable: variability quintile 5 with high expression variability)

From the ATAC-seq datasets (*N* = 3), we identified 89,156 accessible peaks for hemocytes. Of these, 4558 overlapped with promoter regions. Heatmaps for methylated and unmethylated gene bodies show that chromatin accessibility around the transcription start site is more prominent when methylation is low (Additional file 11). Consistent with observations in mouse embryonic stem cells [34], the most expressed genes (quintile 5) had a considerably elevated level of chromatin accessibility (Fig. 4a). We also found low to moderately methylated genes (genes with a mean gene body methylation ratio of 0–0.4) to be distinctly more accessible than highly methylated genes (Fig. 4b). Consistently, housekeeping genes, which are often strongly methylated in marbled crayfish, were found in chromatin states with limited accessibility (Fig. 4c).

In further analyses, we also explored the relationship between gene body methylation, chromatin accessibility and gene expression variability. Consistent with earlier observations in other organisms [25, 26, 35], we observed that low-methylated genes show greater gene expression variability (Fig. 4d). This inverse correlation between gene body methylation and gene expression variability was also conserved in the two other tissues that were analyzed, hepatopancreas and abdominal muscle (Additional file 12). Also, convincing examples for high-methylated genes with low gene expression variability, and low-methylated genes with high expression variability were identified (Additional file 12). We next divided genes into low-methylated (average methylation ratio < 0.4) and high-methylated (average methylation ratio > 0.4) gene to understand the association between gene expression variation and chromatin accessibility. The results showed that low-methylated genes with stable expression were distinctly more accessible than low-methylated genes with high gene expression variability (Fig. 4e). In contrast, high-methylated genes showed no major difference in accessibility for stably and variably expressed genes (Fig. 4f). These findings suggest that gene body methylation promotes stable expression of poorly accessible genes.

### Increased gene body methylation and reduced gene expression variability in *P. fallax*

**Fig. 5:**
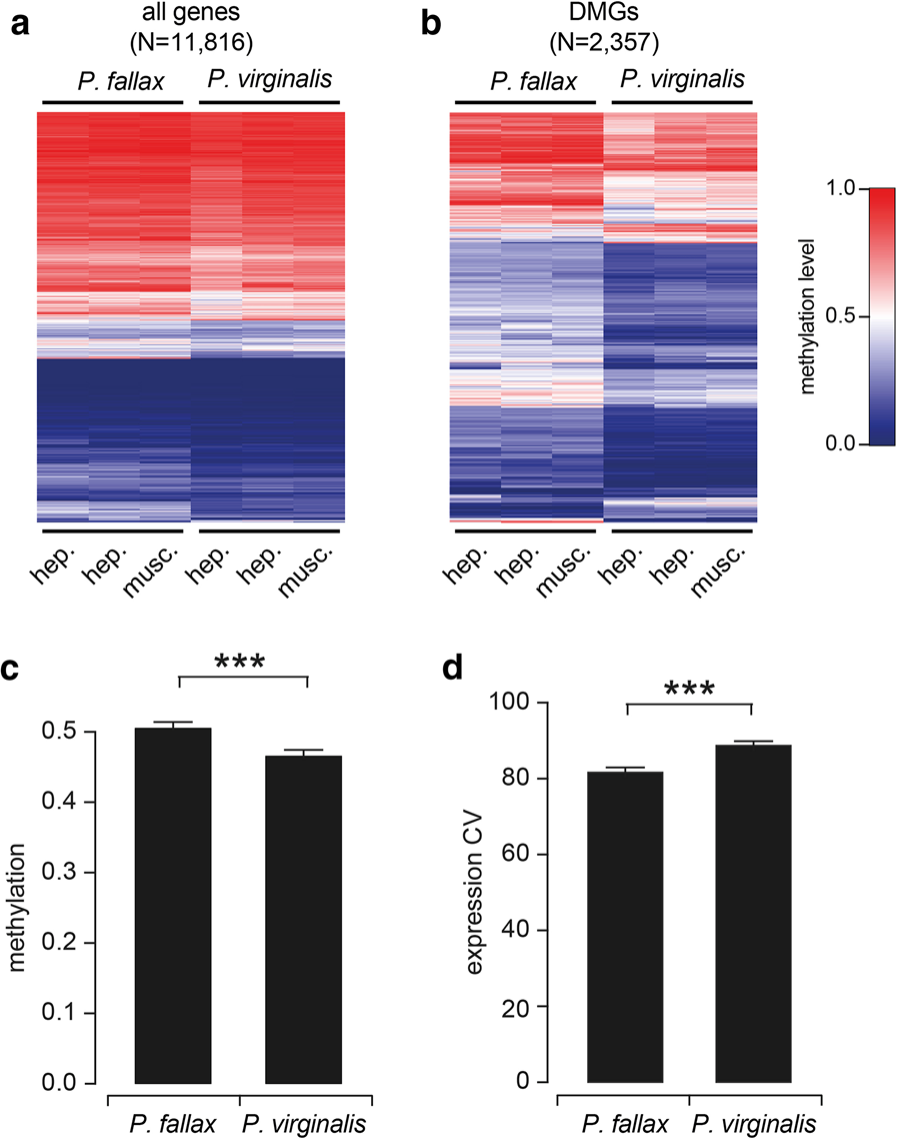
Gene body methylation and gene expression variability in *P. fallax*. **a** Comparative analysis of gene body methylation patterns in *P. virginalis* and *P. fallax*. **b** Species-specific differential gene body methylation. The heatmap shows differentially methylated genes (DMGs) with an average species-specific methylation difference > 0.1. **c** *P. fallax* has a significantly higher average gene body methylation level than *P. virginalis* (*P* < 2.2e^−16^, two-tailed *t* test). **d** Comparison of gene expression variation between marbled crayfish and *P. fallax*. Coefficients of expression variation are indicated for triplicate RNA-seq datasets from the abdominal musculature. The difference between the two species is highly significant (*P* < 5.58e^−13^, two-tailed *t* test)

The marbled crayfish is a recent clonal descendant of the sexually reproducing slough crayfish, *P. fallax* [5, 6]. However, *P. fallax* shows no evidence for invasiveness and populates a defined area in Florida and southern Georgia [36–38]. We therefore generated three *P. fallax* datasets (2 × hepatopancreas, 1 × abdominal musculature), with genome coverage ranging from 10 × to 11 × (Additional file 2) for a comparative analysis of *P. fallax* and marbled crayfish methylomes. As the two species are defined by a very close phylogenetic and genetic relationship [7], reads could be mapped to the marbled crayfish genome. The results revealed a methylation pattern that was overall similar to *P. virginalis* (Fig. 5a). However, we also identified 2357 genes with species-specific methylation differences, the majority of which (> 90%) appeared more methylated in *P. fallax* (Fig. 5b). Overall, gene body methylation levels were significantly reduced in marbled crayfish (Fig. 5c, *P* < 2.2e^−16^), consistent with earlier findings that suggested a moderate but significant reduction in global DNA methylation levels during the transition from *P. fallax* to marbled crayfish [6]. Notably, gene expression variability was significantly elevated in marbled crayfish (Fig. 5d, *P* < 5.58e^−13^). Whether gene body hypomethylation facilitates the phenotypic adaptation of marbled crayfish through increased gene expression variability will have to be addressed in future studies.

## Discussion

DNA methylation is a highly conserved modification in the animal kingdom [14, 15]. However, relatively little is known about its potential function in epigenetic regulation outside of mammals. Our study provides an in-depth analysis of DNA methylation in marbled crayfish, an emerging model organism and new invasive species that is characterized by genetic uniformity and high adaptive potential.

A large number of arthropod methylomes have been published over the past years [14–16]. This includes the methylomes from two crustaceans, *Daphnia* [29] and *P. hawaiensis* [30]. However, all known arthropod methylomes have so far been analyzed using DNA preparations from whole animals or a single, specific tissue. We carried out a direct comparison of arthropod methylation patterns from different tissues and from different animals. Our results show that the marbled crayfish methylome is highly conserved between different tissues and therefore substantially different from paradigmatic mammalian methylomes. Tissue-invariant methylation patterns have also been concluded from a comparison of single sperm and muscle methylomes from the tunicate *Ciona intestinalis* [39]. However, this feature has not been investigated systematically yet and it will be interesting to determine its conservation in invertebrates.

The stability of DNA methylation levels and patterns in marbled crayfish contrasts the dynamic expression of the DNA methylation toolkit during development and in different adult tissues and may suggest additional non-catalytic functions of these enzymes [40]. It should also be noted that the erasure of parental DNA methylation patterns is considered a fundamental requirement for the establishment of totipotency and organismal development in mammals [41, 42]. It is possible that DNA methylation reprogramming in marbled crayfish occurs before the earliest developmental stage that could be investigated by whole-genome bisulfite sequencing (stage 1.7). Alternatively, DNA methylation may not play a major role in the development of the parthenogenetic marbled crayfish.

The marbled crayfish methylome also showed several additional defining features. This includes the relatively sparse methylation of repeats. While similar observations have been made in a few other organisms, including honeybees [43], repeat methylation is a functionally important feature of many animal and plant genomes [44–46]. Also, while mammalian genomes are characterized by dynamic methylation at regulatory regions, such as promoters and enhancers [47–49], the marbled crayfish methylome is characterized by gene body methylation.

**Fig. 6:**
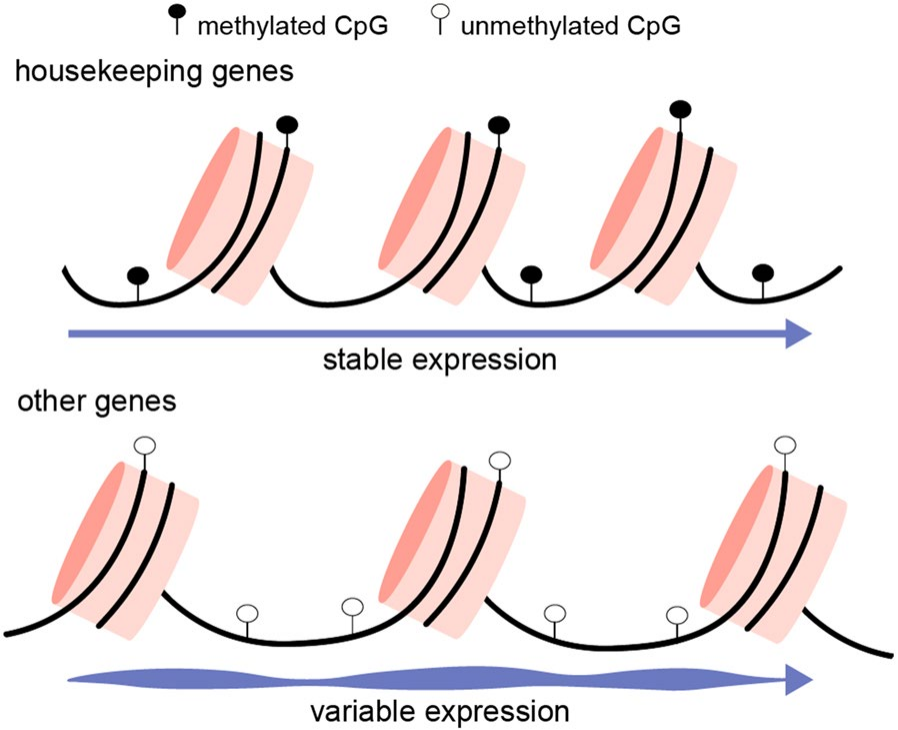
Gene body methylation in marbled crayfish stabilizes gene expression of poorly accessible genes. Housekeeping genes are commonly found in chromatin with limited accessibility and are usually methylated (upper panel). This results in a more stable expression when compared with other genes, which are low-methylated and packaged in more accessible chromatin (lower panel). Black filled circle, methylated CpG. Empty circle, unmethylated CpG

Gene body methylation is often associated with actively transcribed genes [50]. However, we found that gene body methylation in the marbled crayfish does not show a clear correlation with gene expression levels. Our genome-wide chromatin accessibility analysis revealed that the most highly accessible genes were methylated at low levels and most strongly expressed. Similar findings were also recently published in mouse embryonic stem cells [34]. Furthermore, and in agreement with previous observations in insects and in human tissues [25, 26, 35], our analyses showed that gene body methylation inversely correlates with gene expression variability. Finally, the results from our integrative analysis of whole-genome bisulfite sequencing, ATAC-seq and RNA-seq identified two distinct states and shed light on the potential function of gene body methylation (Fig. 6): high-methylated genes (such as housekeeping genes) reside in poorly accessible chromatin and are stably expressed, while low-methylated genes reside in open chromatin and are variably expressed. In this context, gene body methylation might function to increase the specificity of DNA-binding proteins, such as transcription factors [51], in poorly accessible chromatin structures and thus result in a more stable pattern of gene expression.

Of note, we also found a large number of genes to be hypomethylated in marbled crayfish when compared with its parent species, *P. fallax*. In addition, we also observed that gene expression variability was significantly increased in marbled crayfish. Variable gene expression has recently been identified as a key mechanism for coral adaptation to a variable environment [52]. Furthermore, epigenetic variability has been shown to increase fitness in simulations with fluctuating environments [53]. Altogether, our findings thus raise the interesting possibility that hypomethylation of gene bodies and its associated gene expression variability provide a mechanism for the adaptability and invasive potential of marbled crayfish.

## Methods

### Animal culture

Laboratory animals were bred as described before [6] and handled according to institutional guidelines for the care of laboratory animals. Wild animals were collected from lake Moosweiher (Germany, N48°01.844′E07°48.368′), Moramanga (Madagascar, S18°47.350′E48°14.764′) and lake Reilingen (Germany, N49°17.649′E08°32.672′), in compliance with local fishery regulations. Additional information is provided in Additional file 2.

### Identification of *P. virginalis* Dnmt and Tet homologs

*P. virginalis Dnmt1*, *Dnmt3* and *Tet* homologs were identified by using the respective annotated coding sequences from *Daphnia pulex*. Blast (v. 2.2.29 +) was used to align assembled marbled crayfish transcripts against the *Daphnia pulex* sequences. Candidate sequences were validated by searching with Blast against the non-redundant protein sequence database. Read coverage was analyzed by using Bowtie2 (v. 2.2.3) [54] to map transcriptome reads to the respective sequences. Finally, alignments were illustrated in IGV (v. 2.3.34) [55]. In addition, the 3′ SMARTER RACE kit (Takara) was used to resolve remaining ambiguities at the 3′-end of Dnmt3.

### RNA and DNA preparation

Samples of organs and tissues from adult crayfish for DNA and RNA preparation were taken under a dissection microscope, snap-frozen in liquid nitrogen and stored at − 80 °C until extraction of nucleic acids. Embryonated eggs and juveniles were snap-frozen in liquid nitrogen. Genomic DNA was isolated using the Blood and Cell Culture DNA Kit (Qiagen, Hilden, Germany), and total RNA was purified with Trizol (Invitrogen, Darmstadt, Germany).

### Expression analysis

For first-strand cDNA synthesis, RNA was reverse-transcribed using the QuantiTec Reverse Transcription Kit (Qiagen, Hilden, Germany). qRT-PCR analyses were performed on a LightCycler 480 Real-Time PCR System (Roche, Mannheim, Germany) using the Absolute QPCR SYBR Green Mix (Thermo Scientific, St. Leon-Rot, Germany). The expression levels of Dnmt1, Dnmt3 and Tet were determined by the mean crossing point (Cp) value of three technical replicates using the TATA-box-binding protein (TBP) as a reference gene. Primer sequences were as follows: DNMT1_for: GGGAGAAGGCACTGATTGG and DNMT1_rev: CGATCATCGTTGTTCACCAG; DNMT3_for: GAATGGAACATCAGCACCTGC and DNMT3_rev: CGGTGCTCTCATTCCACAATC; Tet_for: CCAGTAGAAGTGATCAACAGTG and Tet_rev: CCTCCAATATCTGGATCGTGG; TBP_for: CCACAGCTACAGAACATCG and TBP_rev: CTCATGATGACGGCTGC.

### Whole-genome bisulfite sequencing

Genomic DNA was isolated as described above. The TruSeq PCR-Free Library Prep Kit (LT; Illumina, San Diego, US) was used for library preparation and the Epitect Kit (Qiagen) for bisulfite conversion. Library amplification was performed using the Kapa HiFi HotStart Uracil + ReadyMix (2 ×; Kapa Biosystems). Samples were then sequenced on an Illumina HiSeq platform.

### ATAC-seq

Approximately 500 µL of hemolymph was collected from three independent animals using a 23-G needle inserted in the abdomen of a cold-anesthetized crayfish. One volume of anticoagulant solution (0.14 M and NaCl, 0.1 M glucose, 30 mM Na_3_ Citrate.2 H_2_O, 26 mM citric acid, 0.5 M EDTA) was added prior to centrifugation for 5 min at 300 x g and 4 °C. After washing the cell pellet twice with sterile and cold PBS 1 ×, 50.000 hemocytes were immediately used for the ATAC library preparation [56]. The transposase reaction was optimized and a 20-min reaction was used to avoid DNA “over transposition.” The subsequent steps were as described in the original protocol [33]. Libraries were sequenced on an Illumina HiSeq platform (Additional file 8).

### Whole-genome bisulfite sequencing data analysis

Read pairs were quality trimmed (minimum quality value ≥ 15 and minimum length ≥ 36 bp), and both marbled crayfish and *P. fallax* data were mapped to the marbled crayfish genome assembly using BSMAP version 2.73 [57]. Correctly mapped read pairs (appropriate orientation and distance to each other) with both reads mapping uniquely to the same scaffold were used for methylation calling. The methylation ratio (methylation calling) for each CpG was determined by the Python script distributed with the BSMAP package. The provided Python script was slightly changed to analyze only reads fulfilling the following additional criteria: (1) minimum quality value of the base at C position ≥ 30 and (2) minimum quality value of the two bases before and after the C position ≥ 20. Only C-positions with a minimum coverage of three reads per strand were used in further analyses. Bisulfite conversion rates and mapping rates are provided in Additional file 2.

Raw data for *P. hawaiensis* and *D. pulex* were downloaded from NCBI using accessions PRJNA306836 and GSE60475, respectively. Custom R scripts were used to determine methylation levels in the genome and genomic features. Violin plots were generated for individual methylomes by R’s ggplot2.violinplot function. Housekeeping genes were identified by blasting human housekeeping genes against the genome assembly. Heatmaps were generated by the heatmap.2 function of R. Data were tested for differential methylation using a Wilcoxon rank-sum test. This was applied to each gene as a paired difference test to see whether the means of the two tissue groups are significantly different from each other. The wilcox.test function in R was used with a *p* value cutoff of 0.1. Barplots for repeat count and methylation were generated in *R* using ggplot2′s geom_bar function.

### RNA-seq data analysis

Rsem [58] was used to calculate expression levels (TPM values) for each sample of the RNA-seq datasets. TPM values were then used to calculate the coefficient of variation of expression levels per tissue and per species, methylation deciles and correlations between the two. For expression ranks, genes were grouped into quintiles and octiles by their TPM values. For hemocytes, correlation of expression levels (TPMs) between samples was confirmed (Additional file 10) and datasets were pooled for gene expression level analyses.

### ATAC-seq data analysis

Raw sequencing data were trimmed and quality filtered (TrimGalore-v0.4.5). Reads were then mapped against the reference genome using Bowtie2 [54], and duplicates were removed with samtools [59]. Broad ATAC peaks were called using MACS2 [60]. After confirming a high correlation between the three biological replicates (Additional file 9), we pooled the three samples for downstream analyses. ATAC-seq coverage was directly extracted from the merged bam file for regions surrounding the transcription start site by using samtools’ bedcov function. Heatmaps and metagene plots were produced using the image function of R and the geom_smooth function of ggplot2.

## Additional files

**Additional file 1.** Housekeeping genes (HKG) expression during different developmental stages and tissues. qRT-PCR was performed using primers to five different HKG, TATA-box-binding protein (TBP), endoribonuclease gene (Dicer1), survival motor neuron protein (Smn), Histone H2A and glyceraldehyde 3-phosphate dehydrogenase (GAPDH).

**Additional file 2.** Whole-genome bisulfite sequencing details.

**Additional file 3.** General characteristics of the marbled crayfish methylome. **a** Logo plot for methylated cytosines. **b** Distribution of the average CpG methylation level (methylation ratio). **c** Strand-specific density of methylated CpGs (mCpG) across the scaffold 48720 (Watson strand: blue, Crick strand: red). The density was calculated by dividing the number of methylated CpGs (methylation ratio ≥ 0.8 and coverage ≥ 3) by the length using a 1-kb non-overlapping sliding window.

**Additional file 4.** Correlation of gene body methylation levels with different gene features. **a** Normalized CpG content [amount of observed CpGs to amount of expected CpGs (o/e)] was classified as low (< 0.6), medium (≥ 0.6, < 1.2) and high (≥ 1.2). **b** Boxplot of average gene methylation by gene length in kb. **c** Predicted marbled crayfish genes were translated into protein sequences and mapped to different phylogenetic nodes with the leftmost representing the oldest and the rightmost the youngest groups.

**Additional file 5.** Methylation of repetitive sequences. The heatmap shows average methylation levels of selected repeat classes in eight independent samples (columns). Only repeats with sufficient coverage in all eight samples are shown. The four most frequent repeat classes are shown (LINEs, SINEs, DNA transposons, LTRs), as well as TcMar-Tigger as an example of a highly methylated repeat class, and rRNAs as an example for a non-transposon repeat class. Methylation levels are indicated on a scale from 0 (blue) to 1 (red). E1.7: stage 1.7 embryos, hep.: hepatopancreas, musc.: abdominal musculature, hem.: hemocytes. Colors denote individual animals.

**Additional file 6.** RNA sequencing details.

**Additional file 7.** Correlation between DNA methylation and gene expression levels. Scatter plots show the promoter methylation (left) and gene body methylation (middle) levels in relationship to gene expression levels. Boxplots (right) show the relationship between gene body methylation and gene expression ranks. Results are shown for all genes (**a**) and for housekeeping genes (**b**).

**Additional file 8.** ATAC sequencing details.

**Additional file 9.** ATAC-seq quality controls showing library quality (top left), pairwise comparisons of ATAC peak intensities from three independent libraries and a representative Genome Browser screen shot of read enrichment (bottom panel).

**Additional file 10.** Reproducibility between three independent hemocyte RNA-seq datasets. Correlation of log10 TPM values for pairwise RNA-seq replicates.

**Additional file 11.** Heatmaps of chromatin accessibility for high-methylated (methylation level > 0.5, left) and low-methylated (methylation level < 0.5, right) genes around transcription start sites (TSS).

**Additional file 12.** Correlation between DNA methylation and gene expression variation in hepatopancreas and abdominal musculature. **a** Low methylation levels correlate with higher gene expression coefficient of variation for hepatopancreas and abdominal musculature. Methylation rank 0 represents completely unmethylated genes. **b** Representative genome browser tracks and expression levels (red bars) for a highly methylated gene with low gene expression variation (top) and a lowly methylated gene with high gene expression variation (bottom).
