# Supplementary figures and images for: The methylome of the marbled crayfish links gene body methylation to stable expression of poorly accessible genes
Source: Epigenetics Chromatin. 2018 Oct 4;11:57. doi: 10.1186/s13072-018-0229-6 (PMC6172769; doi:10.1186/s13072-018-0229-6)

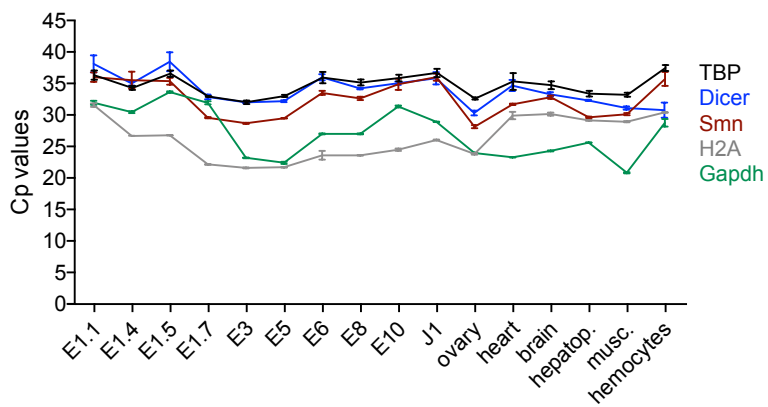

Supplement: Supplementary file 1 — Additional file 1. Housekeeping genes (HKG) expression during different developmental stages and tissues. qRT-PCR was performed using primers to five different HKG, TATA-box-binding protein (TBP), endoribonuclease gene (Dicer1), survival motor neuron protein (Smn), Histone H2A and glyceraldehyde 3-phosphate dehydrogenase (GAPDH). [file 13072_2018_229_MOESM1_ESM.pdf]

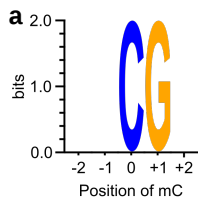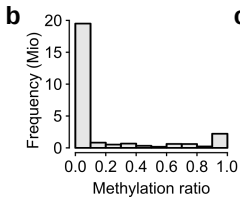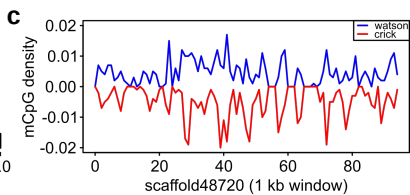

Supplement: Supplementary file 3 — Additional file 3. General characteristics of the marbled crayfish methylome. a Logo plot for methylated cytosines. b Distribution of the average CpG methylation level (methylation ratio). c Strand-specific density of methylated CpGs (mCpG) across the scaffold 48720 (Watson strand: blue, Crick strand: red). The density was calculated by dividing the number of methylated CpGs (methylation ratio ≥ 0.8 and coverage ≥ 3) by the length using a 1-kb non-overlapping sliding window. [file 13072_2018_229_MOESM3_ESM.pdf]

**a**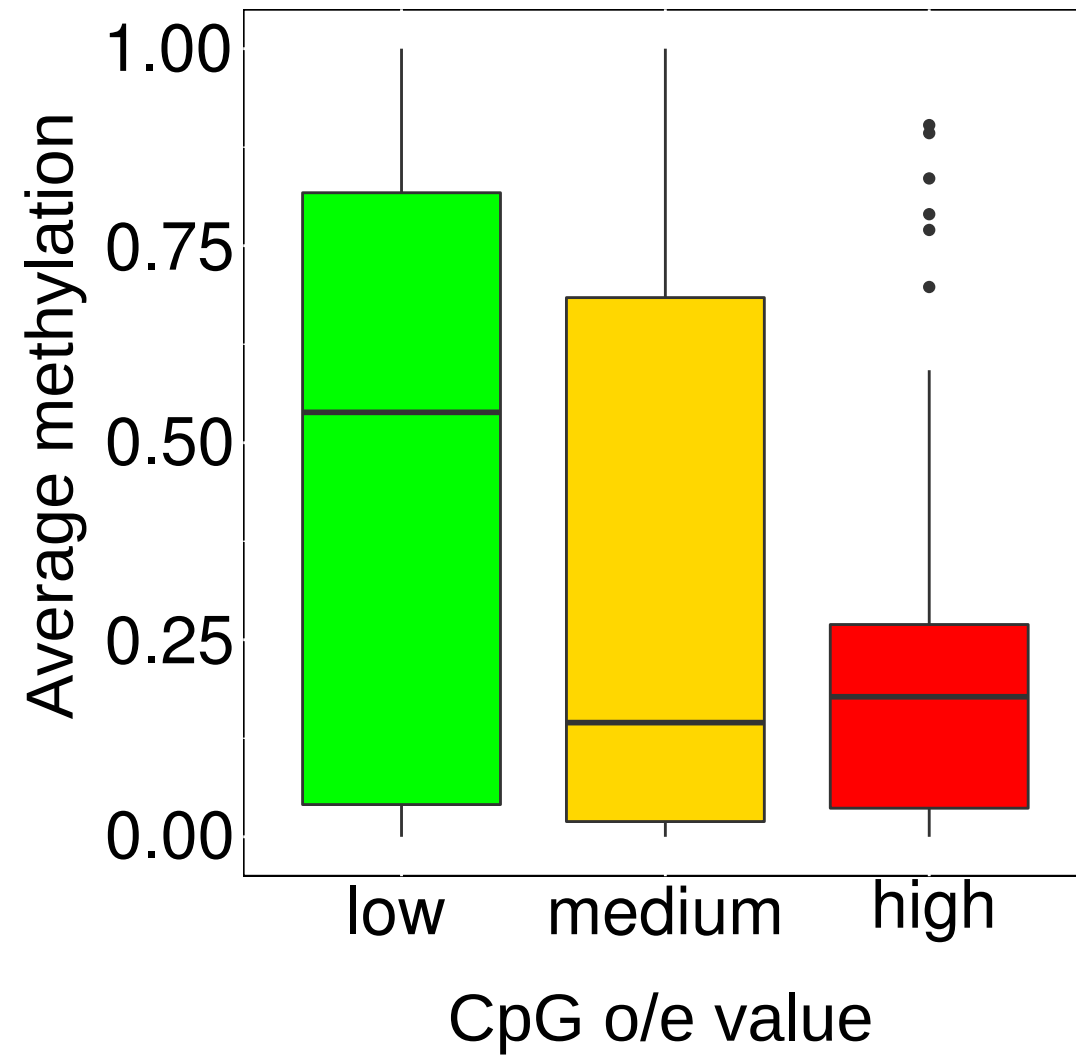**b**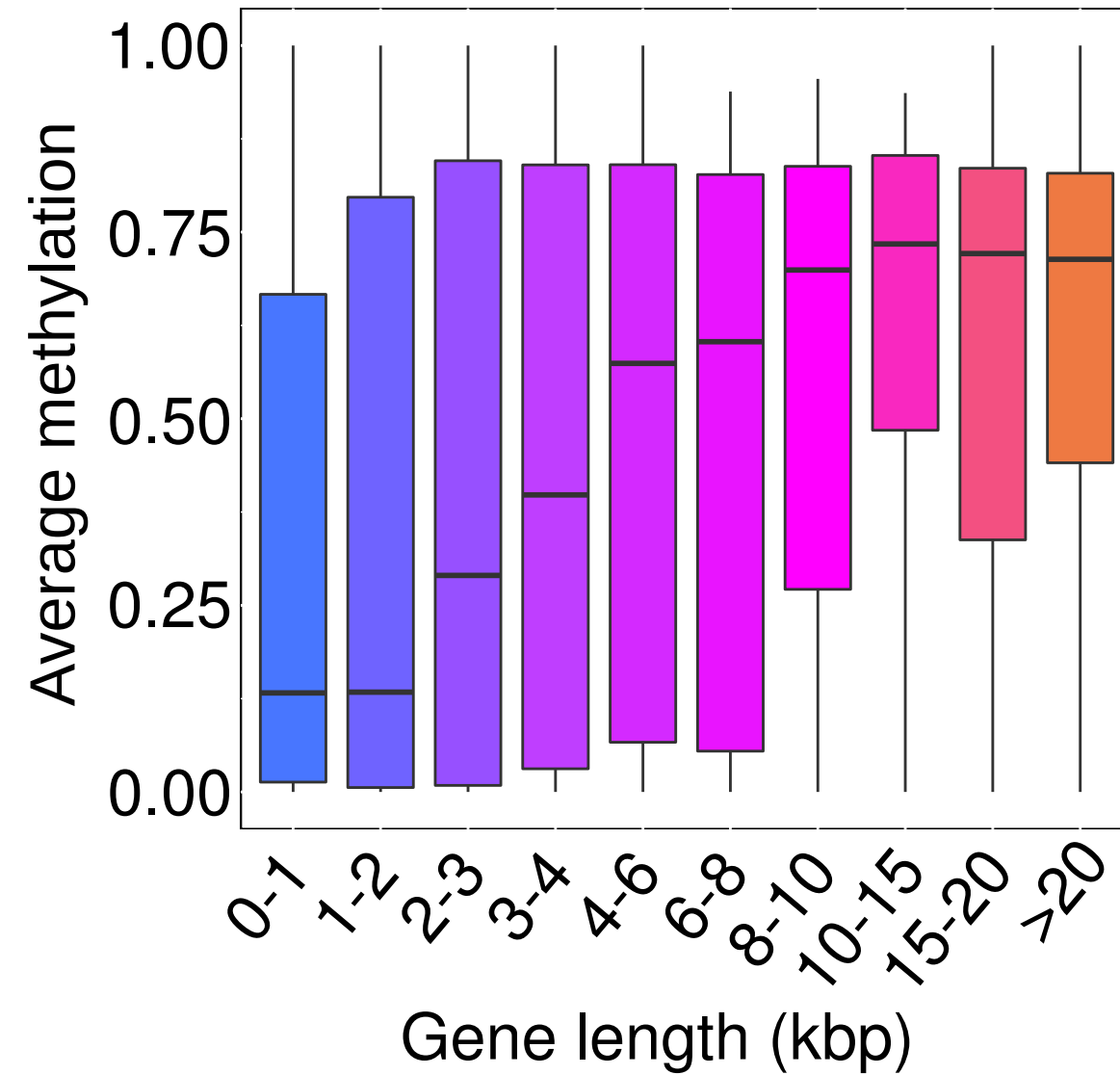**c**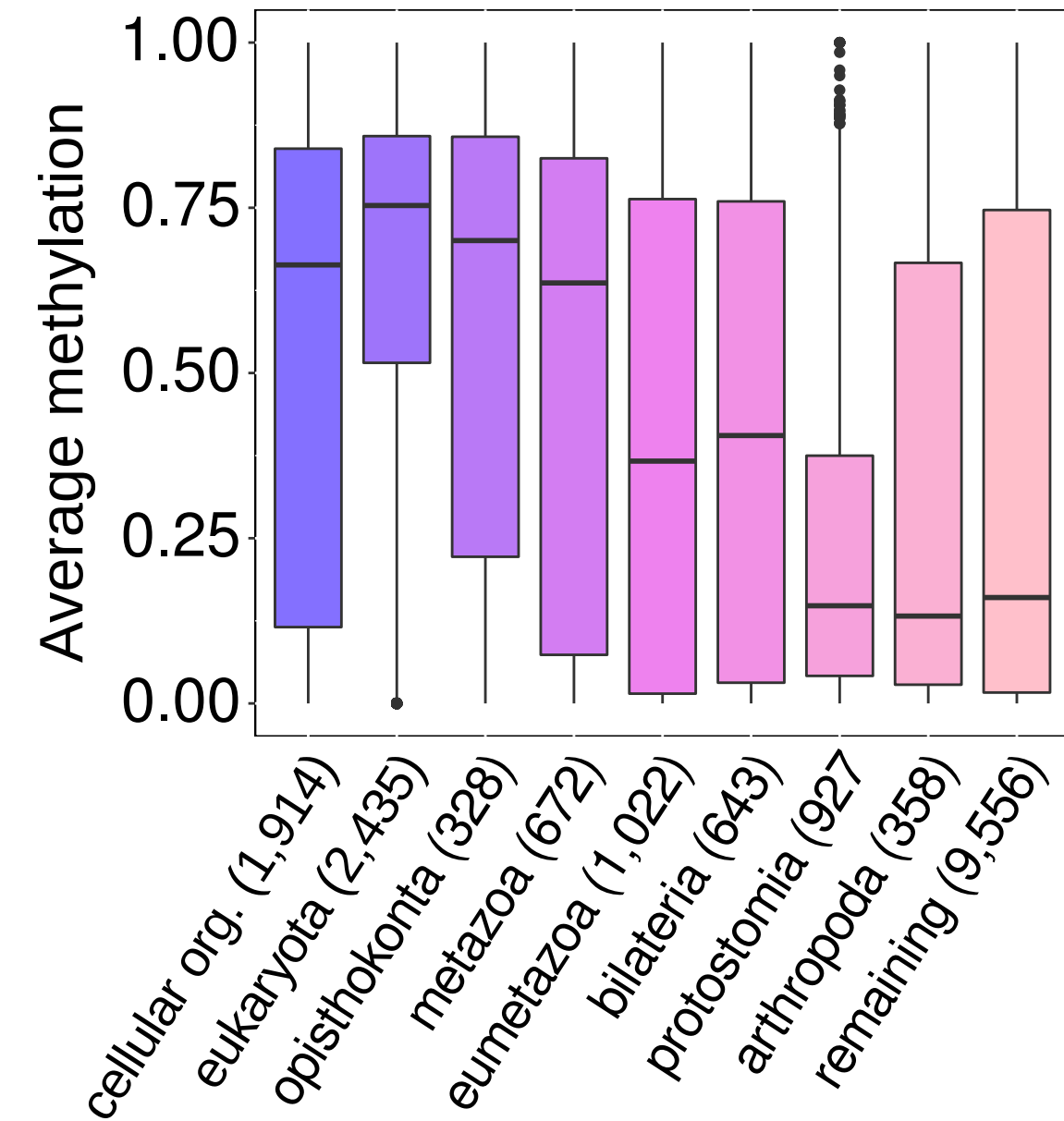

Supplement: Supplementary file 4 — Additional file 4. Correlation of gene body methylation levels with different gene features. a Normalized CpG content [amount of observed CpGs to amount of expected CpGs (o/e)] was classified as low (< 0.6), medium (≥ 0.6, < 1.2) and high (≥ 1.2). b Boxplot of average gene methylation by gene length in kb. c Predicted marbled crayfish genes were translated into protein sequences and mapped to different phylogenetic nodes with the leftmost representing the oldest and the rightmost the youngest groups. [file 13072_2018_229_MOESM4_ESM.pdf]

LINEs  
(N=25,622)

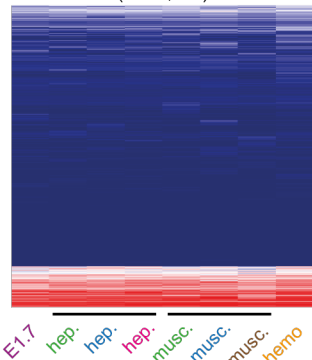

SINEs  
(N=14,821)

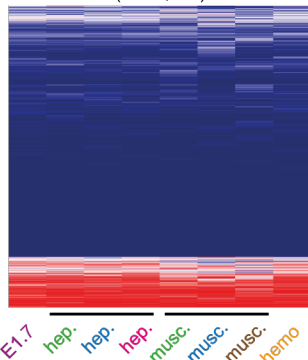

DNA transposons  
(N=7,144)

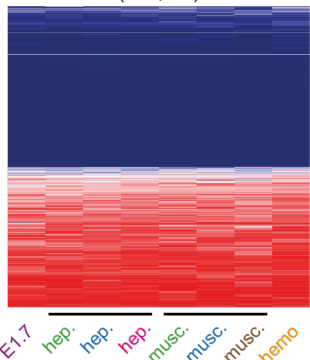

LTRs  
(N=6,483)

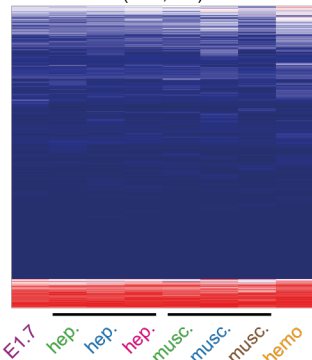

TcMar-Tigger  
(N=2,416)

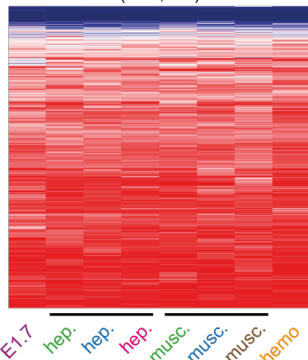

rRNAs  
(N=198)

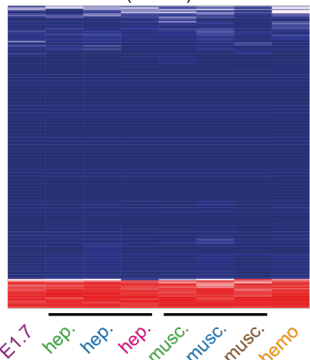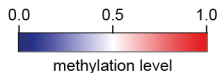

Supplement: Supplementary file 5 — Additional file 5. Methylation of repetitive sequences. The heatmap shows average methylation levels of selected repeat classes in eight independent samples (columns). Only repeats with sufficient coverage in all eight samples are shown. The four most frequent repeat classes are shown (LINEs, SINEs, DNA transposons, LTRs), as well as TcMar-Tigger as an example of a highly methylated repeat class, and rRNAs as an example for a non-transposon repeat class. Methylation levels are indicated on a scale from 0 (blue) to 1 (red). E1.7: stage 1.7 embryos, hep.: hepatopancreas, musc.: abdominal musculature, hem.: hemocytes. Colors denote individual animals. [file 13072_2018_229_MOESM5_ESM.pdf]

**a**

All genes avg prom meth

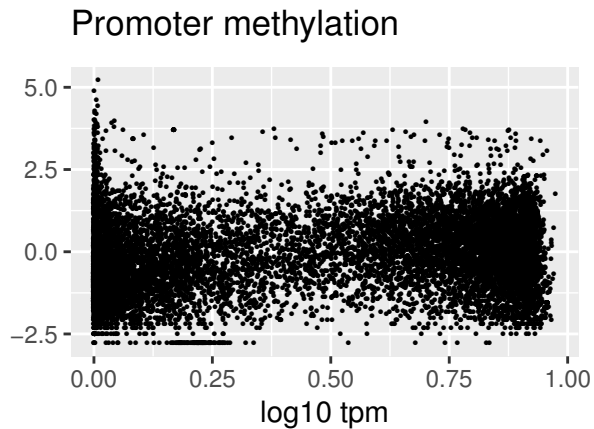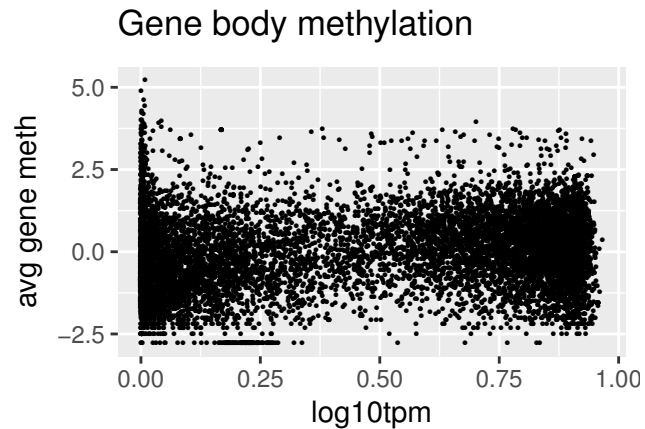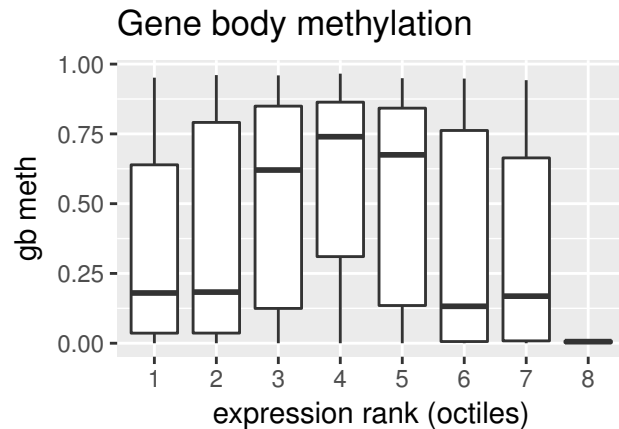**b**

HKGs avg prom meth

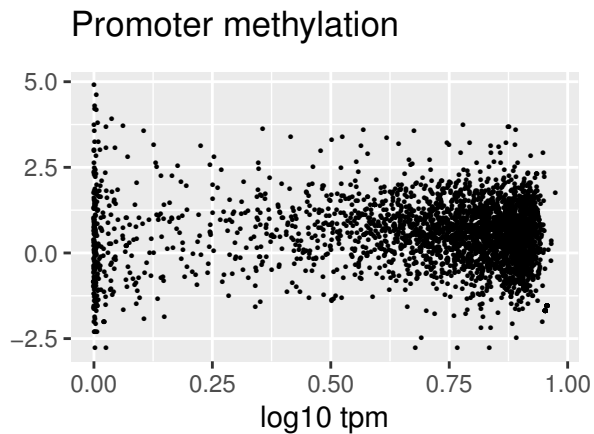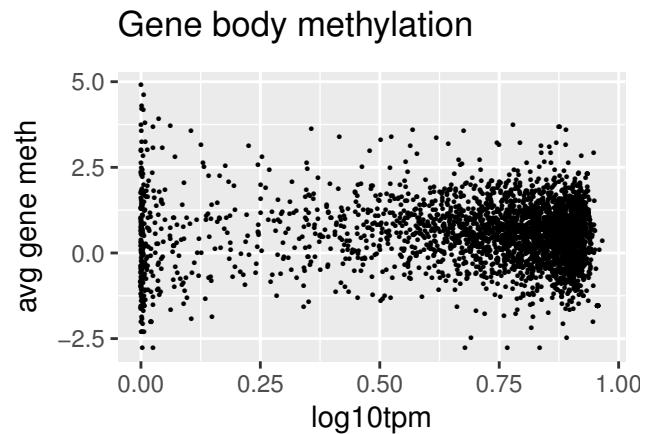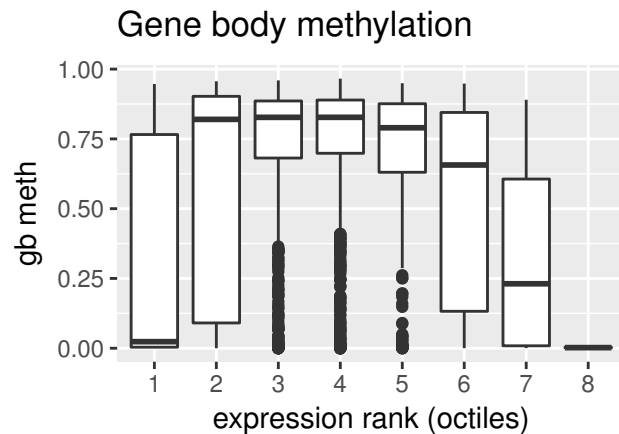

Supplement: Supplementary file 7 — Additional file 7. Correlation between DNA methylation and gene expression levels. Scatter plots show the promoter methylation (left) and gene body methylation (middle) levels in relationship to gene expression levels. Boxplots (right) show the relationship between gene body methylation and gene expression ranks. Results are shown for all genes (a) and for housekeeping genes (b). [file 13072_2018_229_MOESM7_ESM.pdf]

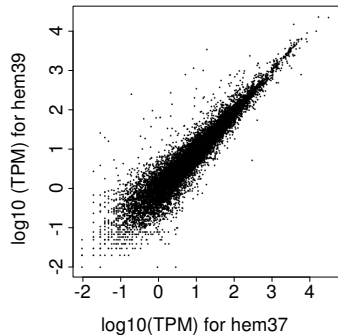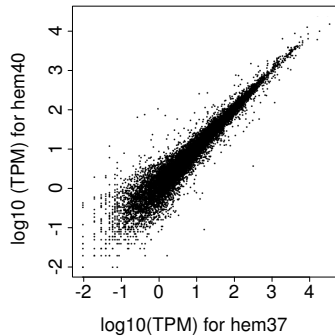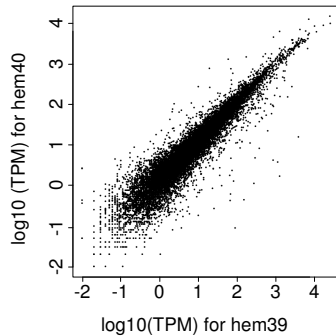

Supplement: Supplementary file 10 — Additional file 10. Reproducibility between three independent hemocyte RNA-seq datasets. Correlation of log10 TPM values for pairwise RNA-seq replicates. [file 13072_2018_229_MOESM10_ESM.pdf]

**a**

methylated genes sorted by accessibility

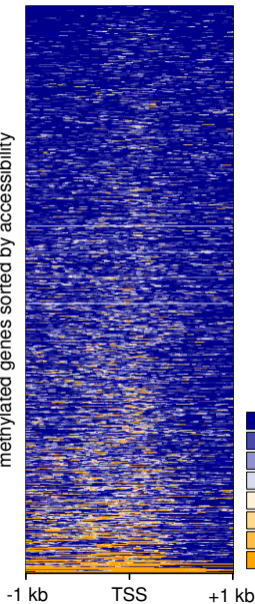**b**

unmethylated genes sorted by accessibility

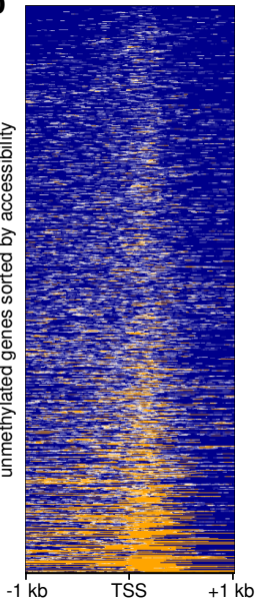

Supplement: Supplementary file 11 — Additional file 11. Heatmaps of chromatin accessibility for high-methylated (methylation level > 0.5, left) and low-methylated (methylation level < 0.5, right) genes around transcription start sites (TSS). [file 13072_2018_229_MOESM11_ESM.pdf]
