# Supplementary material for: The methylome of the marbled crayfish links gene body methylation to stable expression of poorly accessible genes
Source: Epigenetics Chromatin. 2018 Oct 4;11:57. doi: 10.1186/s13072-018-0229-6 (PMC6172769; doi:10.1186/s13072-018-0229-6)
Supplement: Supplementary file 2 — Additional file 2. Whole-genome bisulfite sequencing details. [file 13072_2018_229_MOESM2_ESM.pdf]

Whole-genome bisulfite sequencing details.

ID	species	tissue	source	mapping %	conv. %	genome coverage	% CpGs covered	seq.
Pvir#2	*P. virginalis*	hep.	lab stock	55	99.7	9.8x	79	PE100
Pvir#3	*P. virginalis*	hep.	Moosweiher (Germany)	53	99.8	8.8x	76	PE100
Pvir#6	*P. virginalis*	hep.	lab stock	52	99.2	11.4x	77	PE100
Pvir#2	*P. virginalis*	musc.	lab stock	45	99.6	23.4x	87	PE150
Pvir#3	*P. virginalis*	musc.	Moosweiher (Germany)	50	99.5	20.4x	85	PE150
Mora	*P. virginalis*	musc.	Moramanga (Madagascar)	26	99.3	13.8x	80	PE150
E1.7	*P. virginalis*	E1.7	lab stock	33	99.7	15.2x	81	PE150
hem	*P. virginalis*	hemo.	Reilingen (Germany)	27	99.1	17.9x	81	PE150
Pfal#3	*P. fallax* (female)	hep.	aquarium supply	50	99.9	10.8x	68	PE100
Pfal#4	*P. fallax* (female)	hep.	aquarium supply	51	99.5	10.2x	67	PE100
Pfal#4	*P. fallax* (female)	musc.	aquarium supply	52	99.5	10.8x	67	PE100

Abbreviations: hep.: hepatopancreas, musc.: abdominal musculature, E1.7: embryonic stage 1.7, hemo.: hemolymph, conv.: conversion, seq.: sequencing protocol, PE: paired-end. Baseline mapping ratios are around 50%, due to the high fragmentation level of the reference genome Lower mapping ratios in some of the samples are due to concomitantly sequenced bacterial DNA. Lab stocks and wild catches from Moosweiher have been described in Vogt et al., *Biology Open* 2015, **4**(11):1583-1594.
