# Supplementary material for: The methylome of the marbled crayfish links gene body methylation to stable expression of poorly accessible genes
Source: Epigenetics Chromatin. 2018 Oct 4;11:57. doi: 10.1186/s13072-018-0229-6 (PMC6172769; doi:10.1186/s13072-018-0229-6)
Supplement: Supplementary file 6 — Additional file 6. RNA sequencing details. [file 13072_2018_229_MOESM6_ESM.pdf]

RNA-seq details.

ID	species	tissue	yield [Mbp]	% mapping	seq.
Pvir#2	*P. virginalis*	hepatopancreas	42,032	88	PE100
Pvir#2	*P. virginalis*	abd. musculature	9,144	89	PE100
Pvir#6	*P. virginalis*	hepatopancreas	10,656	90	PE100
Pvir#6	*P. virginalis*	abd. musculature	8,752	92	PE100
Pvir#7	*P. virginalis*	hepatopancreas	11,047	90	PE125
Pvir#7	*P. virginalis*	abd. musculature	4,719	90	PE125
hem37	*P. virginalis*	hemocytes	3,488	85	SE50
hem39	*P. virginalis*	hemocytes	3,231	89	SE50
hem40	*P. virginalis*	hemocytes	3,920	87	SE50
Pfal#1	*P. fallax* (female)	abd. musculature	9,495	90	PE125
Pfal#3	*P. fallax* (female)	abd. musculature	9,161	90	PE125
Pfal#4	*P. fallax* (female)	abd. musculature	8,716	90	PE125

seq.: sequencing protocol
