# Supplementary material for: The methylome of the marbled crayfish links gene body methylation to stable expression of poorly accessible genes
Source: Epigenetics Chromatin. 2018 Oct 4;11:57. doi: 10.1186/s13072-018-0229-6 (PMC6172769; doi:10.1186/s13072-018-0229-6)
Supplement: Supplementary file 8 — Additional file 8. ATAC sequencing details. [file 13072_2018_229_MOESM8_ESM.pdf]

ATAC-seq details.

ID	species	tissue	yield [Mbp]	% mapping	seq.
hem1	*P. virginalis*	hemocytes	12,717	78	PE125
hem2	*P. virginalis*	hemocytes	10,849	73	PE125
hem3	*P. virginalis*	hemocytes	6,423	62	PE125

seq.: sequencing protocol
