# Supplementary material for: The methylome of the marbled crayfish links gene body methylation to stable expression of poorly accessible genes
Source: Epigenetics Chromatin. 2018 Oct 4;11:57. doi: 10.1186/s13072-018-0229-6 (PMC6172769; doi:10.1186/s13072-018-0229-6)
Supplement: Supplementary file 9 — Additional file 9. ATAC-seq quality controls showing library quality (top left), pairwise comparisons of ATAC peak intensities from three independent libraries and a representative Genome Browser screen shot of read enrichment (bottom panel). [file 13072_2018_229_MOESM9_ESM.pdf]

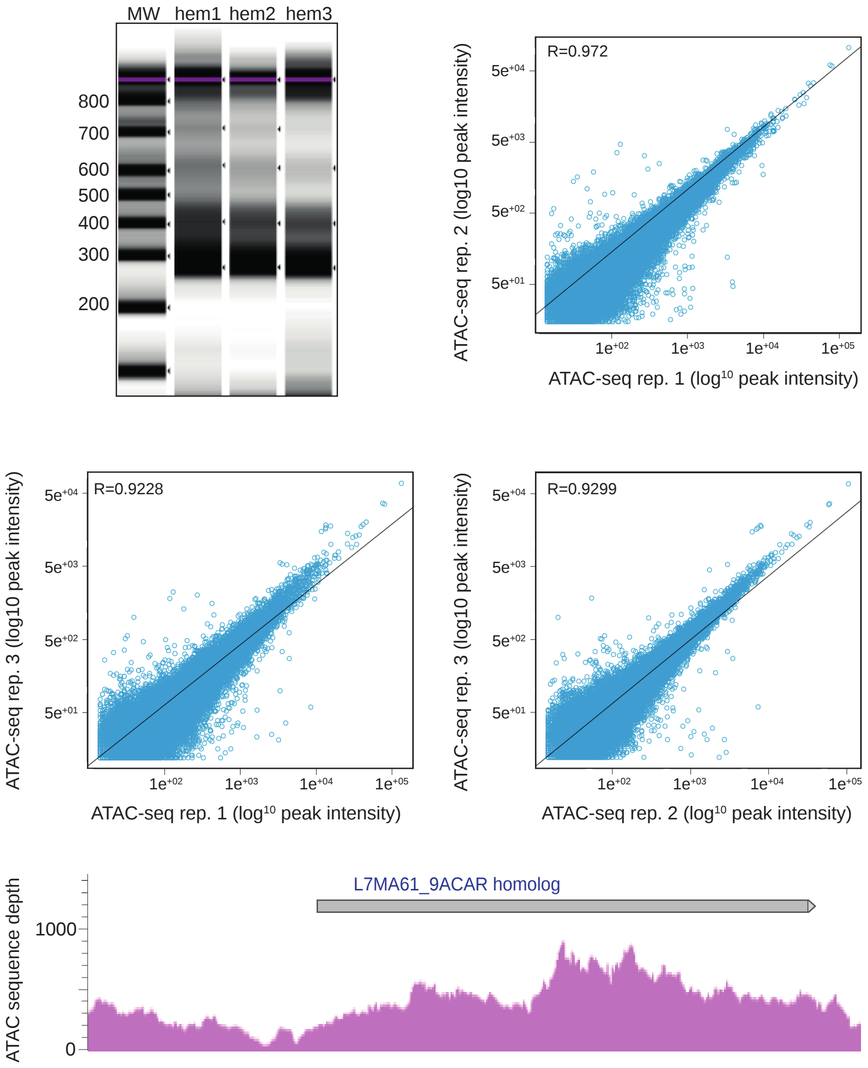

MW
hem1
hem2
hem3
800
700
600
500
400
300
200
R=0.972
ATAC-seq rep. 2 (log10 peak intensity)
ATAC-seq rep. 1 (log10 peak intensity)
5e+04
5e+03
5e+02
5e+01
1e+02
1e+03
1e+04
1e+05
R=0.9228
ATAC-seq rep. 3 (log10 peak intensity)
ATAC-seq rep. 1 (log10 peak intensity)
R=0.9299
ATAC-seq rep. 3 (log10 peak intensity)
ATAC-seq rep. 2 (log10 peak intensity)
L7MA61_9ACAR homolog
ATAC sequence depth
1000
0
