# Supplementary material for: The methylome of the marbled crayfish links gene body methylation to stable expression of poorly accessible genes
Source: Epigenetics Chromatin. 2018 Oct 4;11:57. doi: 10.1186/s13072-018-0229-6 (PMC6172769; doi:10.1186/s13072-018-0229-6)
Supplement: Supplementary file 12 — Additional file 12. Correlation between DNA methylation and gene expression variation in hepatopancreas and abdominal musculature. a Low methylation levels correlate with higher gene expression coefficient of variation for hepatopancreas and abdominal musculature. Methylation rank 0 represents completely unmethylated genes. b Representative genome browser tracks and expression levels (red bars) for a highly methylated gene with low gene expression variation (top) and a lowly methylated gene with high gene expression variation (bottom). [file 13072_2018_229_MOESM12_ESM.pdf]

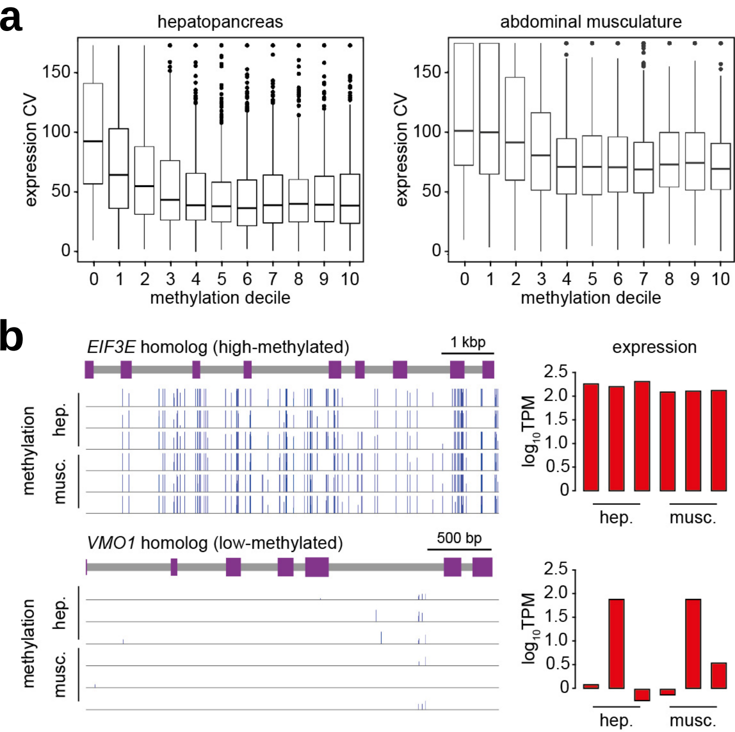
a
hepatopancreas
abdominal musculature
expression CV
methylation decile
150
100
50
0
0 1 2 3 4 5 6 7 8 9 10
b
EIF3E homolog (high-methylated)
1 kbp
methylation
hep.
musc.
expression
log10TPM
2.5
2.0
1.5
1.0
0.5
0
VMO1 homolog (low-methylated)
500 bp
